# Evaluation of land resources carrying capacity based on entropy weight and cloud similarity

**DOI:** 10.1038/s41598-024-59692-2

**Published:** 2024-04-20

**Authors:** Changlin Xu, Li Yang

**Affiliations:** 1 School of Mathematics and Information Science, North Minzu University, Yinchuan, 750021 China; 2 The Key Laboratory of Intelligent Information and Big Data Processing of Ningxia Province, North Minzu University, Yinchuan, 750021 Ningxia China

**Keywords:** Land resource carrying capacity (LRCC), Cloud model, Entropy weight method, Normal cloud similarity, Information technology, Environmental sciences, Environmental social sciences

## Abstract

Land is the foundation of human life and development, which is also the most important part of a country. The study of land carrying capacity is one of the important contents of land management, wherein the evaluation of land resource carrying capacity (LRCC) is an important reference for land resource planning. Aiming at the information fuzziness and uncertainty in the evaluation of LRCC, firstly, a comprehensive evaluation model based on entropy weight and normal cloud similarity was proposed, which is based on cloud model theory and combined with normal cloud similarity measurement method and entropy weight method. Secondly, taking the asphalt pavement experiment as an example for empirical analysis, the experimental results are consistent with the actual situation, which proves the feasibility and effectiveness of the proposed model. Finally, taking China’s Chongqing city as the research area, the proposed evaluation model is used to study LRCC. The research results indicate that the comprehensive carrying capacity and average carrying capacity of various systems in China’s Chongqing have been improved in the past decade. Among them, the comprehensive carrying capacity rose from the second level during the "12th Five-Year Plan" period to the third level during the "13th Five-Year Plan" period. In the future, it is necessary to focus on the improvement of soil and water resources system and economic and technological system. This conclusion reflects LRCC of Chongqing in China objectively and has a reference value for Chongqing's land planning.

## Introduction

Land is an important carrier of resource environment and economic development, which is also a necessary condition for human life. As the city speeds up the industrialization process and experiences a growing population, the increasingly prominent contradiction between humans and land becomes evident. As an important index to evaluate the utilization of urban land resources and the comprehensive development level of society, the research of land resources carrying capacity (LRCC) are becoming more and more abundant^1–4^. He et al.^5^ use the technique for order preference by similarity to an ideal solution (TOPSIS) to evaluate LRCC of China’s Anhui Province from 2006 to 2015, and the development trend of LRCC in the next five years is predicted by GM (1,1) model. Sun et al.^6^ applied error back propagation (EBP) to land management for the first time, and found that there is a direct relationship between urbanization and LRCC, then evaluated the carrying capacity of urban land resources. Zhou et al.^7^ conducted a single factor evaluation of the carrying capacity of arable and ecological land from the perspective of relative LRCC, obtained the relative conditions of various provinces and regions in China. It can be seen from the above literature that there are certain uncertainties and randomness in the evaluation research of LRCC, and the data of some evaluation indicators are quite different, leading to large deviations in the evaluation results. As a model to transform qualitative concept and quantitative representation, cloud model can overcome the uncertainty and randomness of LRCC evaluation research and effectively evaluate the evaluation object. The cloud model is a two-way cognitive model between a certain qualitative concept expressed in linguistic values and its quantitative representation, which is used to reflect the uncertainty of concepts in natural language and transform the qualitative concept into a set of quantitative values. It can describe randomness and fuzziness in uncertain information well, and provides a new method for the study of uncertain artificial intelligence. Liu et al.^8^ propose a decision analysis method using uncertain artificial intelligence, and portray the conceptual cloud model of influencing factors through cloud transformation to calculate the cloud weight of each factor. Finally, the comprehensive evaluation results through the cloud model are obtained. Jia et al.^9^ constructed a comprehensive evaluation method based on cloud models and applied it to the renewable capacity of watershed water resources. However, these two methods both use fuzzy numbers or fuzzy decision analysis for evaluation. Once the membership function in the fuzzy evaluation is expressed accurately, it will not have fuzziness. Liu et al.^10^ propose an improved multi-criteria decision making (MCDM) model, which combines swarm fuzzy entropy and cloud model, and uses TOPSIS to sort and evaluate alternative schemes. Zou and Tian^11^ construct primary and secondary weight sets in view of the investment risks and existing uncertainties of CCS projects, and propose an investment risk assessment model based on improved comprehensive cloud to obtain reasonable results. However, the construction of weight sets is relatively complicated. The above literatures use cloud model to deal with fuzziness problems and achieve good results. In order to increase the objectivity of index weight, entropy weight method is a good weight determination method. In the combined application of entropy weight method and cloud model, Gong^12^ uses entropy weight-normal cloud model to build evaluation index system, and uses entropy weight method to calculate the weight of each evaluation index, and then uses normal cloud model to achieve evaluation level division. At the same time, this model is used to evaluate and analyze the urban ecological risk in Hexi region. Guan et al.^13^ take the Huaihe River Basin as the research object, and use the cloud model to select some indicators, so then construct the comprehensive evaluation index system. The entropy weight method was used to calculate the weight of each index. The composite fuzzy meta-model was used to calculate the comprehensive index. Chen et al.^14^ introduced the qualitative and quantitative transformation normal cloud by combination weighting model method, and made a comprehensive evaluation of urban ecological environment quality according to the similarity level and ranking on the basis of considering shape and distance.

At present, cloud model evaluation is mainly based on membership degree to judge the evaluation level. There is few research on cloud evaluation using similarity degree. Cloud model similarity can be well integrated into cloud comprehensive evaluation. As for the study on the similarity measurement of cloud models, Zhang et al.^15^ randomly select cloud droplets based on Monte Carlo idea and calculate the similarity between cloud models by calculating the distance of these cloud droplets. However, the time complexity of the algorithm to calculate the distance between cloud droplets is too high, and the stability of the experimental results is easy to be affected by the number and threshold of cloud droplets. Zhang et al.^16^ regarded the three numerical characteristics of a cloud model as a vector, and constructed the cosine value of the two cloud concepts to discriminate the similarity between two cloud concepts, so then a likeness comparing method based on cloud model (LICM) was proposed. However, the LICM method assigns the same weight to the three numerical characteristics, which results in the poor discrimination ability of this method. Gong et al.^17^ treated normal cloud models as normal fuzzy numbers and used combing fuzzy similarity measure (CFSM) to calculate the ratio of the overlapping area and the non-overlapping area of two cloud models, and then calculates the similarity by the arithmetic mean minimum closeness degree. Because this method involves the calculation of integral, the computational complexity is relatively high. After that, a position-shape-based cloud model (PSCM) method was proposed, in which the PSCM method divides the cloud similarity into shape similarity and position similarity^18^, and uses the numerical characteristics to obtain the final cloud similarity by combining the shape similarity and position similarity. Considering the overall geometric characteristics of cloud model and the contribution of micro-cloud drops, Dai et al.^19^ proposed a cloud model uncertainty similarity measurement method based on the envelope area and the contribution degree of cloud drops for cloud model (EACCM), and carried out the simulation experiments. The EACCM method has comprehensive consideration and high stability, but also high computational complexity.

Based on the above literature research, it can be found that cloud similarity is rarely used in LRCC evaluation at present. However, LRCC has fuzziness and randomness, and some evaluation indicator data have significant differences, which makes the determination of evaluation indicator weights more crucial. The existing LRCC is difficult to solve the problems of ambiguity and subjectivity in determining indicator weights. In this regard, the paper utilizes the characteristics of cloud models with combining entropy weight method and cloud model similarity, thus a comprehensive evaluation model based on entropy weight and normal cloud similarity is proposed. The proposed method effectively solves some problems existing in the evaluation methods of land resource carrying capacity, and also provides a new method for subsequent land research. Let Chongqing in China as the research area to gain a deeper understanding of the development and changes in LRCC. The main work is as follows: (1) Combining the similarity of the normal cloud with the entropy weight, the objective characteristics of the entropy weight are used to calculate the index weight. Calculating the similarity between the evaluated clouds and standard clouds by using similar measurement, and proposing a comprehensive evaluation model based on entropy weight-cloud similarity. (2) Taking the asphalt pavement experiment as an example for empirical analysis to verify the feasibility and effectiveness of the proposed method. The experimental results show that the final results are consistent with the actual situation. (3) Applying the proposed method into the comprehensive evaluation of the Chongqing's LRCC during 2011–2020, and obtaining the corresponding conclusions.

The rest of this article is organized as follows: "Theory and methods" section introduces the main content of normal cloud model and Wasserstein distance, and briefly explains the concept similarity measurement method of normal cloud based on Wasserstein distance. In "Evaluation model" section, a comprehensive evaluation model based on entropy weight and normal cloud similarity is proposed, and the specific evaluation steps are shown. The asphalt pavement is taken as a case to verify the proposed method. "Case analysis" section applies the proposed method into the comprehensive evaluation of Chongqing's in China LRCC, and evaluates the carrying capacity level. The last part is summary and expectation.

## Theory and methods

### Cloud model concept and characteristic curve

#### Definition 1.

^20^ Let *U* be a universal set described by precise numbers, and *C* be the qualitative concept related to *U*. If there is a number $x \in U$, which randomly realizes the concept *C,* and the membership degree of $x$ for *C,* that is, $\mu_{C} (x) \in [0,1][0,1]$, is a random value with steady tendency:

$$ \mu_{C} (x):U \to [0,1],\quad \forall x \in U:x \to \mu_{C} (x), $$

then the distribution of $x$ on *U* is s defined as a cloud, and each $x$ is defined as a cloud drop, noted $Drop(x,\mu_{C} (x))$.

The cloud model generally consists of three numerical characteristics (expectation $Ex$, entropy $En$ and hyper‐entropy $He$) to describe the uncertainty information as a whole, where $Ex$ reflects the central value of uncertainty information; $En$ reflects the degree of discreteness of the data to the expected value $Ex$, and represents the range of the data, that is, reflects the ambiguity of the data; hyper‐entropy ($He$) is the entropy of entropy($En$), which represents the range of random distribution of cloud droplets, reflects the randomness of data, and indicates the degree of discreteness of cloud droplets.

If the distribution of $x$ on *U* satisfies: $x\sim N(Ex,En^{{\prime}{2}} )$, where $En^{\prime}\sim N(En,He^{2} )$, and the degree of certainty on *C* is:

1 $$ \mu (x) = \exp \left\{ { - \frac{{(x - Ex)^{2} }}{{2En{\prime}^{2} }}} \right\}. $$

Then the distribution of $x$ on* U* is called normal cloud.

The expectation curve of normal cloud with entropy was defined by literature^17^ based on the conclusion that the expectation of normal cloud droplet is $Ex$, variance is $En^{2} + He^{2}$ given in literature^21^.

#### Definition 2

^17^ If the random variable $x\sim N(Ex,En^{{\prime}{2}} )$, where $En^{\prime}\sim N(En,He^{2} )$, and $En \ne 0$, then

2 $$ y(x) = {\text{exp}}\left\{ { - \frac{{(x - Ex)^{2} }}{{2(En^{2} + He^{2} )}}} \right\} $$

is called the entropy-containing expectation curve of a normal cloud.

### Introduction to Wasserstein distance

#### Definition 3

^22^ Let $\mu$,$v$ be the measures on the probability space $\Re^{n}$, $\forall x,y \in \Re^{n} ,$ define the *p*-Wasserstein distance as

3 $$ W_{P} (\mu ,v) = \left( {\mathop {\inf }\limits_{\gamma \in \prod (\mu ,v)} \smallint_{{\Re^{n} \times \Re^{n} }} d(x,y)^{p} {\text{d}}\gamma (x,y)} \right)^{1/p} , $$

where $\prod (\mu ,v)$ is the set of joint probability measures $\gamma$ on $\Re^{n} \times \Re^{n}$, the edge distribution of this joint probability distribution are $\mu$ and $v$, $d(x,y)$ is any distance on $\Re^{l}$, $p \ge 1$.

#### Definition 4

^23^ For two multidimensional normal distributions $P_{1}$ and $P_{2}$, the Wasserstein distance is:

4 $$ W(P_{1} ,P_{2} ) = \sqrt {\left\| {m_{1} - m_{2} } \right\| + tr(M_{1} ) + tr(M_{2} ) - 2tr[(\sqrt {M_{1} } M_{2} \sqrt {M_{1} } )^{1/2} ]} , $$

where $m_{1}$ and $m_{2}$ are the mean vectors of $P_{1}$ and $P_{2}$ respectively, $M_{1}$ and $M_{2}$ are the covariance matrices of $P_{1}$ and $P_{2}$ respectively.

According to formula (4), if for two one-dimensional normal distributions $X$ and $Y$ , the Wasserstein distance $d(X,Y)$ between the two is.

5 $$ d(X,Y) = \sqrt {(\mu_{1} - \mu_{2} )^{2} + (\sqrt {\sigma^{2}_{1} } - \sqrt {\sigma^{2}_{2} } )^{2} } , $$

where $\mu_{1}$ and $\mu_{2}$ are the mean of $X$ and $Y$ respectively, $\sigma^{2}_{1}$ and $\sigma^{2}_{2}$ are the variances of $X$ and $Y$ respectively.

### Normal cloud similarity calculation method based on Wasserstein distance

Through the above research, Wasserstein distance and normal cloud similarity are combined to obtain the method of normal cloud similarity based on Wasserstein distance.

#### Definition 5

^24^ Let two normal cloud concepts: $C_{1}$ = $(Ex_{1} ,En_{1} ,He_{1} )$, $C_{2}$ = $(Ex_{2} ,En_{2} ,He_{2} )$, then the similarity measures of $C_{1}$ and $C_{2}$ normal clouds based on Wasserstein distance are

6 $$ sim(C_{1} ,C_{2} ) = {\text{e}}^{{ - d(C_{1} ,C_{2} )}} . $$

where $d(C_{1} ,C_{2} ) = \sqrt {(Ex_{1} - Ex_{2} )^{2} + (\sqrt {En_{1}^{2} + He_{1}^{2} } - \sqrt {En_{2}^{2} + He_{2}^{2} } )^{2} } .$ The larger the $sim(C_{1} ,C_{2} )$, the higher the similarity of the two normal clouds, and the opposite is true. Meanwhile, $sim(C_{1} ,C_{2} )$ also satisfies the following properties.

## Evaluation model

The determination of weights is one of the important factors in comprehensive evaluation. Entropy weight can objectively reflect the weight of indicators and eliminate human interference in the weight of each indicator, thereby making the results more realistic. Based on the advantages of normal cloud similarity in comprehensive evaluation, a comprehensive evaluation model based on entropy weight and normal cloud similarity is proposed by utilizing the characteristics of cloud models in dealing with fuzziness and uncertainty, and combining the objectivity and adaptability advantages of entropy weight method.

### Entropy weight method

Entropy weight method is an objective weighting method. The weight is determined by the information of each index, which can avoid the deviation caused by human subjective factors. The main steps are as follows^25^:

1. According to the initial data set of *n* evaluation indexes of *m* evaluation objects, and established the eigenvalue matrix *X*: $X = (x_{ij} )_{m \times n} (i = 1,2,...,m;j = 1,2,...,n)$.
2. As the dimensions and orders of magnitude of each index are different, it is necessary to standardize the matrix. The processing formulas of positive and negative indicators are as follows:
  7 $$ r_{ij} = (x_{ij} - x_{j}^{\min } )/(x_{j}^{\max } - x_{j}^{\min } ); $$
  8 $$ r_{ij} = (x_{j}^{\max } - x_{ij} )/(x_{j}^{\max } - x_{j}^{\min } ); $$

In the above formula, $x_{j}^{\min }$ and $x_{j}^{\max }$ are respectively the minimum and maximum values of the same index $x_{j}$ in different objects. The normalized matrix is $R = (r_{ij} )_{m \times n} (i = 1,2,...,m;j = 1,2,...,n)$.

1. The entropy of each evaluation index is defined as:
  $$ h_{j} = - \frac{1}{\ln m}\sum\limits_{i = 1}^{m} {f_{ij} \ln } f_{ij} , $$
  where $$f_{ij} = (1 + r_{ij} )/\sum\limits_{i = 1}^{m} {(1 + r_{ij} )}$$.
2. The entropy weight $w_{j}$ of the* j* evaluation index is:
  9 $$ w_{j} = g_{j} /\sum\limits_{j = 1}^{n} {g_{j} } , $$
  where $$g_{j} = 1 - h_{j}$$.

### Construction of evaluation model

According to 2.3 and 3.1, this paper proposes a comprehensive evaluation model based on entropy weight and normal cloud similarity. The steps as follows:

*Step 1*: Establish the factor domain $U = \{ u_{1} ,u_{2} ....,u_{n} \}$ of the evaluation object and the comment domain $V = \{ v_{1} ,v_{2} ....,v_{m} \}$.

*Step 2*: Use entropy weight method to calculate the index weight $W = \{ w_{1} ,w_{2} ....,w_{i} \}$.

*Step 3*: Build a cloud model of the evaluation object. According to the original data under each index $i$, use the backward cloud generator to obtain the cloud concept $A_{i} = (Ex_{i} ,En_{i} ,He_{i} )$ $i = 1,2,...,n$ under each indicator $i$.

*Step 4*: Construct the classification level of the evaluation object. Let the upper and lower boundary values of level $j(j = 1,2,...,m)$ corresponding to index $i(i = 1,2,...,n)$ be $x_{ij}^{1}$ and $x_{ij}^{2}$, then the qualitative concept of level $j$ corresponding to index $i$ can be represented by normal cloud concept, where^26^:

10 $$ Ex_{ij} = (x_{ij}^{1} + x_{ij}^{2} )/2, $$

As $x_{ij}^{1}$ and $x_{ij}^{2}$ are critical values from one evaluation level interval to another evaluation level interval, they are boundary values with randomness and fuzziness, so $x_{ij}^{1}$, $x_{ij}^{2}$ can belong to two adjacent evaluation levels at the same time. Thus, $x_{ij}^{1}$, $x_{ij}^{2}$ has the same membership degree in the two adjacent evaluation levels, namely:

$$ \exp \left\{ { - \frac{{(x_{ij}^{1} - x_{ij}^{2} )^{2} }}{{8(En_{ij} )^{2} }}} \right\} = 0.5, $$

From the upper formula:

11 $$ En_{ij} = (x_{ij}^{1} - x_{ij}^{2} )/2.355. $$

Hyper‐entropy $He_{ij}$ is the entropy of entropy, which determines the degree of dispersion between cloud droplets. The larger the value is, the better the cohesiveness between cloud droplets will be. When the value decreases to 0, the normal cloud concept will degenerate into a normal cloud curve. Hyper‐entropy is generally obtained through experience.

*Step 5*: According to the similarity calculation formula (formula (6)), calculated the similarity between the cloud concept $A_{i} = (Ex_{i} ,En_{i} ,He_{i} )$ of each index $i$ and the evaluation level, and obtained the normal cloud similarity matrix $Z = (z_{ij} )_{n \times m}$.

*Step 6*: Weight set $W = \{ w_{1} ,w_{2} ....,w_{i} \}$ and normal cloud similarity matrix $Z = (z_{ij} )_{n \times m}$ are used for aggregation to obtain the comprehensive similarity matrix* D* at each level.

12 $$ D = W \cdot Z = (d_{1} ,d_{2} ,...d_{m} ), $$

where $d_{j} = \sum\limits_{i = 1}^{n} {w_{i} } z_{ij} ,j = 1,2...m$. According to the principle of maximum similarity, the *j* evaluation level corresponding to the maximum similarity is selected as the result of comprehensive evaluation.

**Figure 1:**
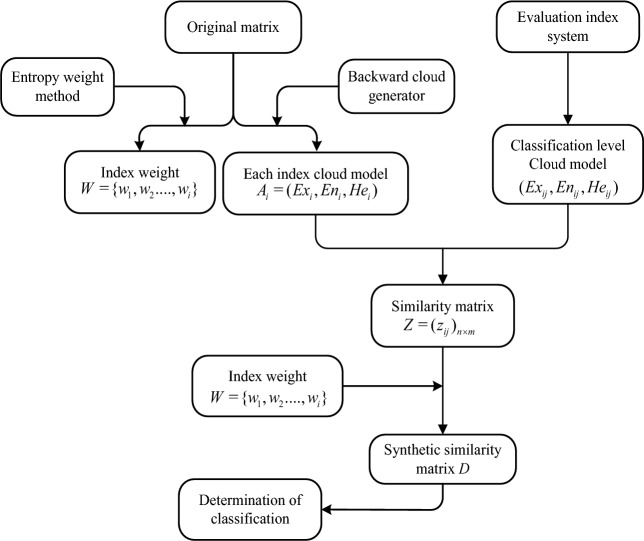
Flow chart of comprehensive evaluation based on entropy weight and normal cloud similarity.

The evaluation process is shown in Fig. 1.

### Validation analysis

In order to verify the feasibility and effectiveness of the proposed method, this section will analyze the similarity measurement method and the comprehensive evaluation model based on entropy weight and normal cloud similarity. In terms of similarity, the classification accuracy and time complexity of WCM have been verified and explained in literature^24^, which will not be elaborated here. In terms of the model, this paper selects the asphalt pavement performance evaluation experiment^27^ as an example for simulation experiments, and compares the results with the original text to demonstrate the accuracy of the model.

Select Xinjiang's S215 Line Sanchakou-Shache Expressway 233 km in length, and with the K81–K86 total 5 km as the test section. Taking the test data in 2016 as the sample and shown in Table 1, establish the entropy weight and normal cloud similarity concept, select five evaluation indexes, and the index evaluation levels were shown in Table 2^27^.

**Table 1:** Damage index detection data of Sanchakou-Shache expressway (2016).

Number	Index	Road section				
		K81-K82	K82-K83	K83-K84	K84-K85	K85-K86
1	PCI	82.34	86.05	83.41	85.54	85.56
2	RDI	84.07	90.25	87.95	74.24	84.18
3	SRI	85.97	89.88	85.45	83.44	87.69
4	PSSI	88.73	87.34	85.92	86.14	83.52
5	RQI	85.71	85.26	84.68	82.41	84.54

**Table 2:** Performance evaluation index of asphalt pavement of expressway.

Index	Level				
	Very high	High	Medium	Low	Very low
PCI	90–100	80–90	70–80	60–70	0–60
RDI	90–100	80–90	70–80	60–70	0–60
SRI	90–100	80–90	70–80	60–70	0–60
PSSI	90–100	80–90	70–80	60–70	0–60
RQI	90–100	80–90	70–80	60–70	0–60

Because the evaluation indicators are positive indicators, Calculate the weight of each evaluation index from Table 1 and formula (7), (9). Meanwhile, according to Table 1, cloud concept $A_{i} = (Ex_{i} ,En_{i} ,He_{i} )$ corresponding to each index is generated through backward cloud generator. The results are shown in Table 3.

**Table 3:** Weight of indicators and corresponding cloud concept of this section.

	PCI	RDI	SRI	PSSI	RQI
Weight	0.221	0.177	0.233	0.196	0.173
Cloud concept $A_{i}$	(84.58,1.71,0.56)	(84.14,4.99,3.54)	(86.49,2.31,0.76)	(86.33,1.71,0.89)	(84.52,1.06,0.70)

The numerical characteristic values (*Ex, En, He*) of each evaluation index are obtained by formula (10) and formula (11), The results are shown in Table 4.

**Table 4:** Digital characteristics of cloud concept for evaluation levels of each index of asphalt pavement.

Index	Level				
	Very high	High	Medium	Low	Very low
PCI	(95,4.246,0.1)	(85,4.246,0.1)	(75,4.246,0.1)	(65,4.246,0.1)	(30,4.246,0.1)
RDI	(95,4.246,0.1)	(85,4.246,0.1)	(75,4.246,0.1)	(65,4.246,0.1)	(30,4.246,0.1)
SRI	(95,4.246,0.1)	(85,4.246,0.1)	(75,4.246,0.1)	(65,4.246,0.1)	(30,4.246,0.1)
PSSI	(95,4.246,0.1)	(85,4.246,0.1)	(75,4.246,0.1)	(65,4.246,0.1)	(30,4.246,0.1)
RQI	(95,4.246,0.1)	(85,4.246,0.1)	(75,4.246,0.1)	(65,4.246,0.1)	(30,4.246,0.1)

According to formula (6), calculate the normal cloud similarity between cloud concept $A_{i}$ corresponding to each index in Table 3 and each evaluation level in Table 4. The normal cloud similarity matrix is shown in Table 5.

**Table 5:** Normal cloud similarity matrix.

Level	Index				
	PCI	RDI	SRI	PSSI	RQI
Very high	0.0000	0.0000	0.0002	0.0001	0.0000
High	0.0834	0.1271	0.0955	0.0691	0.0490
Medium	0.0001	0.0001	0.0000	0.0000	0.0001
Low	0.0000	0.0000	0.0000	0.0000	0.0000
Very low	0.0000	0.0000	0.0000	0.0000	0.0000

Finally, the weight of each index and the normal cloud similarity matrix are used for aggregation, and the comprehensive similarity matrix *D* is calculated according to formula (12). The results are shown in Table 6.

**Table 6:** Comprehensive similarity of asphalt pavement performance of Sanchakou-Shache expressway.

Level	very high	high	medium	low	very low
Comprehensive similarity	0.0001	0.0852	0.0000	0.0000	0.0000

It can be seen from Table 6 that the maximum value of the comprehensive similarity corresponds to the level of high. According to the principle of maximum similarity, the evaluation level of this section is high. This conclusion is consistent with the results in literature^27^ and the actual situation. In the original literature, the entropy weight method is also used to determine the index weight to ensure relative objectivity. The difference between this paper and literature^27^ is that comprehensive similarity is used in this paper to determine the evaluation level, while comprehensive certainty is used in the original literature, but the results obtained are consistent. This fully demonstrates the accuracy and effectiveness of the comprehensive evaluation model proposed in this paper based on entropy weight and normal cloud similarity.

## Case analysis

After the successful establishment of the comprehensive evaluation model, this paper applies the model to the evaluation of Chongqing's LRCC. The application characteristics of the model are illustrated by practical cases. Meanwhile, the evaluation of LRCC in Chongqing can also provide reference for the local land planning and development.

As the foundation of human development, land provides a variety of resources for human development and guarantees the progress of human society. In recent decades, the explosive growth of population has brought about the overdevelopment of land and the sharp consumption of resources, and land resources are facing unprecedented pressure. With the increasingly severe situation of land resources, the evaluation of LRCC has been developed rapidly. In the 12th and 13th Five-Year plans for national development, ecological and environmental protection has been given an important position, and it is proposed to continuously strengthen ecological and environmental protection and improve ecological and environmental quality. In the "13th Five-Year Plan", the construction of ecological civilization is elevated to the national strategy, highlighting the status of ecological civilization construction. LRCC refers to the limit of the scale and intensity of various human activities that land resources can carry in a certain period, a certain spatial area and under certain economic, social, resource and environmental conditions^28–30^.

Chongqing is located in the southwest of China, upstream of the Yangtze River. The permanent population is over 30 million, and the terrain is mainly hilly and mountainous. Chongqing is a municipality directly under the central government, a national central city, and a core city of the Chengdu Chongqing Economic Circle in China. It holds an important strategic position in the southwest region and throughout the country. This section takes Chongqing as the research area and uses the "12th Five Year Plan" and "13th Five Year Plan" as time standards to evaluate the comprehensive LRCC and the carrying capacity of various systems in Chongqing at the standard layer. This study chooses China’s Chongqing as the research area, examining its geographical and strategic location, attempting to reveal the patterns of population, resources, and environment changes in Chongqing, and providing relevant references for its subsequent development.

### Data source

The data in this section are mainly from China Statistical Yearbook (2011–2021), Chongqing Statistical Yearbook (2011–2021), Chongqing Social and economic development Announcement, and some data are from news reports, etc.

### Evaluation index determination

The research on LRCC at home and abroad has been very mature, and rich experience has been accumulated in the selection of evaluation indicators and the construction of index system. The evaluation indicators is the basis of research, and the selection of indicators should follow the principles of science, hierarchy, feasibility, independence, dynamism and regionality^31^.

In this section, based on the land resource utilization situation in Chongqing and referring to previous research and achievements^30,31^, LRCC evaluation index system including 12 indexes was constructed from four criterion layers: water and soil resource system, ecological environment system, social and cultural system, and economic and technological system. As shown in Table 7.

**Table 7:** Evaluation index system of LRCC.

Criterion layer	Index layer	Units	Property
Water and soil resource system	Per capita cultivated area $P_{1}$	acres/per capita	+
	Per capita food resources $P_{2}$	kg/per capita	+
	Per capita water resources $P_{3}$	m^3^/per capita	+
Ecological environment system	Forest coverage $P_{4}$	%	+
	Good air quality rate $P_{5}$	%	+
	Per capita green space $P_{6}$	m^2^/per capita	+
Social and cultural system	Natural population growth rate $P_{7}$	‰	−
	Urbanization rate $P_{8}$	%	−
	Urban registered unemployment rate $P_{9}$	%	−
Economic and technological system	Disposable income of urban residents $P_{10}$	Ten thousand yuan/per capita	+
	GDP per capita $P_{11}$	Ten thousand yuan/per capita	+
	Local fixed asset investment $P_{12}$	Hundred million yuan/km^2^	+

### Evaluation level division

The classification of level should reflect the level of LRCC and distinguish the influence of different indicators in the evaluation results. In this section, based on relevant national standards, technical specifications and policies, as well as cloud model theory, obtained the numerical characteristics of cloud concepts by formulas (10) and (11). Among them, level I indicates that the carrying capacity level of land resources is very low, level II indicates that the carrying capacity is low, level III indicates that the carrying capacity is medium, level IV indicates that the carrying capacity is high, level V indicates that the carrying capacity is very high^32^. The results are shown in Table 8.

**Table 8:** Numerical characteristics of cloud concept of LRCC index.

Index	Level				
	I	II	III	IV	V
$P_{1}$	(0.40,0.34,0.001)	(0.90,0.09,0.001)	(1.10,0.09,0.001)	(1.39,0.16,0.001)	(1.58,0.16,0.001)
$P_{2}$	(175,148.62,1)	(375,21.23,1)	(425,21.23,1)	(475,21.23,1)	(500,21.23,1)
$P_{3}$	(250,212.32,2)	(750,212.32,2)	(1500,424.63,2)	(2500,424.63,2)	(30,000,424.63,2)
$P_{4}$	(10,8.50,0.005)	(27.5,6.37,0.005)	(45,8.49,0.005)	(65,8.49,0.005)	(75,8.49,0.005)
$P_{5}$	(15,12.74,0.005)	(37.5,6.37,0.005)	(52.5,6.37,0.005)	(67.5,6.37,0.005)	(75,6.37,0.005)
$P_{6}$	(2.5,2.12,0.05)	(6,0.85,0.05)	(7.5,0.42,0.05)	(9,0.85,0.05)	(10,0.85,0.05)
$P_{7}$	(11.5,1.27,0.01)	(8.5,1.27,0.01)	(6.0,0.85,0.01)	(4.0,0.85,0.01)	(1.5,1.27,0.01)
$P_{8}$	(92.5,6.37,0.1)	(77.5,6.37,0.1)	(62.5,6.37,0.1)	(47.5,6.37,0.1)	(20.0,16.99,0.1)
$P_{9}$	(5.35,0.30,0.01)	(4.15,0.30,0.01)	(3.95,0.30,0.01)	(3.30,0.25,0.01)	(1.50,1.27,0.01)
$P_{10}$	(1.0,0.85,0.05)	(2.5,0.43,0.05)	(3.5,0.43,0.05)	(4.5,0.43,0.05)	(5.0,0.43,0.05)
$P_{11}$	(1.25,1.06,0.01)	(3.75,1.06,0.01)	(6.25,1.06,0.01)	(8.75,1.06,0.01)	(10.00,1.06,0.01)
$P_{12}$	(0.15,0.127,0.001)	(0.4,0.085,0.001)	(0.65,0.127,0.001)	(0.9,0.085,0.001)	(1,0.085,0.001)

### Model calculation

This section obtains the index data of Chongqing from 2011 to 2020 through 4.2. The data are shown in Table 9.

**Table 9:** Evaluation index data of Chongqing from 2011 to 2020.

Year	Index											
	$P_{1}$	$P_{2}$	$P_{3}$	$P_{4}$	$P_{5}$	$P_{6}$	$P_{7}$	$P_{8}$	$P_{9}$	$P_{10}$	$P_{11}$	$P_{12}$
2011	1.25	361.41	1747.64	39.00	54.50	17.01	6.54	55.00	3.50	1.85	3.47	0.10
2012	1.24	356.49	1603.06	41.00	55.20	17.41	1.00	56.60	3.30	2.10	3.92	0.11
2013	1.22	350.42	1583.81	42.10	56.40	17.10	4.67	58.30	3.40	2.31	4.35	0.14
2014	1.21	342.99	2111.33	43.10	67.40	16.54	5.10	59.70	3.50	2.51	4.83	0.16
2015	1.19	342.36	2093.08	45.00	80.00	16.10	4.01	61.50	3.60	2.72	5.25	0.19
2016	1.15	346.69	1944.95	45.40	82.20	16.18	5.76	63.30	3.70	2.96	5.83	0.21
2017	1.13	343.53	1924.19	46.50	83.00	16.43	− 1.09	65.00	3.40	3.22	6.41	0.21
2018	1.07	341.22	1657.34	48.30	86.60	16.55	3.38	66.60	3.30	3.49	6.85	0.23
2019	0.88	337.28	1644.50	50.10	86.60	16.16	2.80	68.20	2.60	3.79	7.43	0.24
2020	0.87	337.00	2389.90	52.50	91.00	16.16	− 1.42	69.50	4.50	4.00	7.83	0.25

In order to further analyze the changes of comprehensive and system LRCC in Chongqing in recent ten years. In this paper, the 2011–2020 period of Chongqing is divided into the "12th Five-Year Plan" and the "13th Five-Year Plan", namely 2011–2015 and 2016–2020. According to the property of each indicator, the data in Table 9 are standardized, and use the entropy weight method to calculate the weights of indicators from 2011–2015 to 2016–2020. The results are shown in Table 10.

**Table 10:** Weight of indicators during the 12th Five-Year Plan and 13th Five-Year Plan of Chongqing.

Year	Index											
	$P_{1}$	$P_{2}$	$P_{3}$	$P_{4}$	$P_{5}$	$P_{6}$	$P_{7}$	$P_{8}$	$P_{9}$	$P_{10}$	$P_{11}$	$P_{12}$
2011–2015	0.072	0.101	0.122	0.069	0.11	0.073	0.072	0.076	0.073	0.075	0.078	0.079
2016–2020	0.106	0.090	0.086	0.076	0.078	0.116	0.079	0.073	0.058	0.073	0.07	0.095

The 12th Five-Year Plan (2011–2015) is taken as an example for analysis. According to the data in Table 9, the cloud concept corresponding to each index is generated through backward cloud generator. The results are shown in Table 11.

**Table 11:** Cloud concepts corresponding to each index from 2011 to 2015.

Index	$P_{1}$	$P_{2}$	$P_{3}$	$P_{4}$
Cloud concept	(1.22,0.02,0.01)	(350.74,8.24,1.22)	(1827.78,275.15,94.34)	(42.04,2.05,0.93)
Index	$P_{5}$	$P_{6}$	$P_{7}$	$P_{8}$
Cloud concept	(62.70,11.03,0.71)	(16.83,0.51,0.03)	(4.26,1.76,1.04)	(58.22,2.43,0.77)
Index	$P_{9}$	$P_{10}$	$P_{11}$	$P_{12}$
Cloud concept	(3.46,0.11,0.03)	(2.30,0.32,0.10)	(4.36,0.68,0.20)	(0.14,0.04,0.01)

According to formula (6), the similarity between the normal clouds corresponding to each index and the normal clouds of each evaluation level is calculated, and the similarity matrix shown in Table 12.

**Table 12:** Similarity matrix.

Level	Index											
	$P_{1}$	$P_{2}$	$P_{3}$	$P_{4}$	$P_{5}$	$P_{6}$	$P_{7}$	$P_{8}$	$P_{9}$	$P_{10}$	$P_{11}$	$P_{12}$
I	0.4143	0.0000	0.0000	0.0000	0.0000	0.0000	0.0007	0.0000	0.1497	0.2479	0.0435	0.9132
II	0.7191	0.0000	0.0000	0.0000	0.0000	0.0000	0.0135	0.0000	0.4897	0.8024	0.4921	0.7664
III	0.8730	0.0000	0.0000	0.0000	0.0000	0.0001	0.1214	0.0032	0.5926	0.2996	0.1467	0.5948
IV	0.8056	0.0000	0.0000	0.0000	0.0012	0.0004	0.2933	0.0000	0.8080	0.1104	0.0123	0.4662
V	0.6818	0.0000	0.0000	0.0000	0.0000	0.0011	0.0567	0.0000	0.1025	0.0670	0.0035	0.4219

The weight of each index during 2011–2015 and the normal cloud similarity matrix are used for aggregation. According to formula (12), the comprehensive similarity matrix is calculated. The results are shown in Table 13.

**Table 13:** Comprehensive similarity matrix from 2011 to 2015.

Level	I	II	III	IV	V
Comprehensive similarity	0.1349	0.2475	0.1964	0.1849	0.0996

According to the principle of maximum similarity, it is concluded that the comprehensive LRCC of Chongqing during the 12th Five-Year Plan period (2011–2015) is II, that is, the low carrying capacity level. Similarly, the comprehensive similarity matrix of the 13th Five-Year Plan period (2016–2020) can be obtained by using the same method. The results are shown in Table 14.

**Table 14:** Comprehensive similarity matrix from 2016 to 2020.

Level	I	II	III	IV	V
Comprehensive similarity	1.1987	1.9666	2.7678	1.7295	1.1225

Also, according to the principle of maximum similarity, it is concluded that LRCC in Chongqing during 2016–2020 is level III, that is the medium carrying capacity level. The conclusion indicates that Chongqing's LRCC has been improved during the 13th Five-Year Plan period, and Chongqing has considered the protection of ecological environment while developing economy.

In addition, in order to further analyze the factors affecting LRCC, this section takes the "12th Five-Year Plan" and "13th Five-Year Plan" as the time nodes to analyze the water and soil resources system, ecological environment system, social and cultural system, economic and technological system. Similarly, calculate the comprehensive similarity matrix for 2011–2015 and 2016–2020. the results are shown in Tables 15 and 16.

**Table 15:** Comprehensive similarity matrix of criterion layer from 2011 to 2015.

System	Level				
	I	II	III	IV	V
Water and soil resource system	0.1015	0.1762	0.2139	0.1976	0.1672
Ecological environment system	0.0000	0.0000	0.0003	0.0007	0.0003
Social and cultural system	0.0498	0.1666	0.2370	0.3635	0.0525
Economic and technological system	0.4057	0.6856	0.3487	0.1986	0.1665

**Table 16:** Comprehensive similarity matrix of criterion layer from 2016 to 2020.

System	Level				
	I	II	III	IV	V
Water and soil resource system	0.1969	0.3279	0.3394	0.2604	0.2154
Ecological environment system	0.0000	0.0000	0.0004	0.0003	0.0008
Social and cultural system	0.0420	0.1307	0.1577	0.1882	0.0862
Economic and technological system	0.3730	0.4566	0.7106	0.3585	0.2638

**Figure 2:**
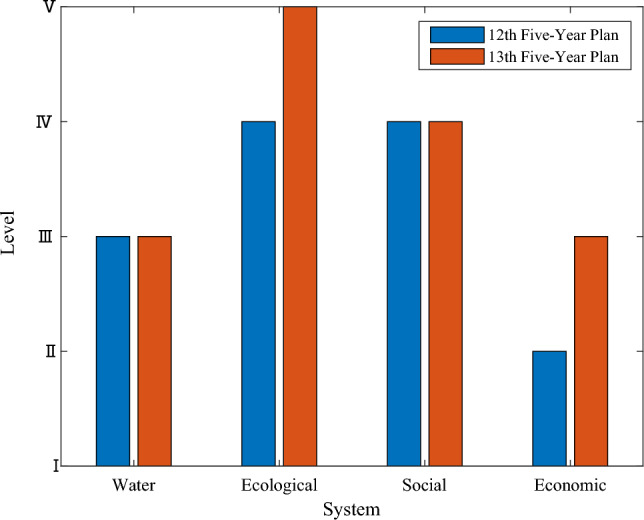
The carrying capacity levels of each system during the 12th Five-Year Plan and 13th Five-Year Plan periods.

The data in Tables 15 and 16 were visualized to compare LRCC levels of each system in the two-time stages, where the Water and soil resource system was abbreviated as Water, Ecological environment system was abbreviated as Ecological, Social and cultural system was abbreviated as Social, Economic and technological system was abbreviated as Economic. As shown in Fig. 2.

From Fig. 2, during the "12th Five-Year Plan" and "13th Five-Year Plan" period, in terms of water and soil resources system, the carrying capacity level was level III, that is the medium carrying capacity level. This indicates that the system carrying capacity of water and soil resources in Chongqing has been maintained at a relatively stable level in the past ten years. With the growth of Chongqing's population, the intensity of land development is also increasing, which will have a certain impact on the comprehensive LRCC. In terms of the ecological environment system, the carrying capacity during the "12th Five-Year Plan" period is level IV, that is, the high carrying capacity level, while that of the "13th Five-Year Plan" period is level V, that is, the very high carrying capacity level. This change indicates that the ecological environment of Chongqing has development in the past five years. From the perspective of indicators, the forest coverage rate and air quality excellence rate are constantly improving, which have a positive impact on carrying capacity. As far as the environment is concerned, Chongqing is located in southwest China and the upper reaches of the Yangtze River. Most of its administrative areas are hilly and mountainous. Therefore, it has natural advantages in urban greening and well protected ecological environment system. In terms of social and humanistic system, the carrying capacity during the "12th Five-Year Plan" and "13th Five-Year Plan" are both level IV, that is, the high carrying capacity level. In recent years, with the rapid development of economy, the urbanization rate of Chongqing is constantly increasing, and the unemployment rate and the natural growth rate of population are declining. These factors are affecting the change of social carrying capacity. In terms of economic and technological system, it has been uplevel from the low level in the 12th Five-Year Plan to the medium level in the 13th Five-Year Plan, which indicates that Chongqing's economy has been effectively developed in these five years. Since the Western Development and the construction of the Chengdu Chongqing Economic Circle, Chongqing's economy has developed rapidly, and people's income levels have also improved. The improvement of these indicators directly drives the improvement of the carrying capacity of the economic and technological system.

In general, while developing economy, Chongqing still takes the protection of ecological environment. To further improve LRCC in the future, Chongqing can start from soil and water resources system, economic and technological system, and make efforts to prepare land planning and economic construction.

## Conclusions

In this paper, a comprehensive evaluation model based on entropy weight and normal cloud similarity is proposed, which based on the cloud model and the objective characteristics of entropy weight method. The empirical analysis is carried out by taking the asphalt pavement experiment as an example. The conclusion is consistent with the original literature and the actual situation, that shows the feasibility and effectiveness of the proposed method. Finally, the model is applied to the evaluation of Chongqing's LRCC from 2011 to 2020, and the comprehensive carrying capacity and the system carrying capacity of Chongqing are analyzed. The research shows that the comprehensive LRCC of Chongqing has been improved from level II to level III. The bearing capacity of each system has also been improved. Relatively speaking, the land and water resources system, and the economic and technological system still need to be further developed. This study is realistic and objective, and can provide some reference for Chongqing's future land use planning.

## Discussion

This paper still has some shortcomings and needs improvement in the following aspects, which need to be further explored in the subsequent research:

1. The construction of the index system can add more evaluation indicators and make the evaluation system more diversified.
2. When determining index weights, subjective methods such as analytic hierarchy process and expert scoring can be combined with objective methods to combine and assign weights, so as to make the determination of weights more comprehensive and scientific, and further comprehensively evaluate the carrying capacity of land resources.

## Data Availability

The datasets used and/or analyzed during the current study are available from the corresponding author upon reasonable request.
